# Supplementary material for: Paper-Based Exosomal MicroRNA-21 Detection for Wound Monitoring: A Proof of Concept and Clinical Validation Trial Study
Source: Int J Mol Sci. 2023 Jun 6;24(12):9822. doi: 10.3390/ijms24129822 (PMC10298464; doi:10.3390/ijms24129822)
Supplement: Supplementary file 1 [file ijms-24-09822-s001.zip › ijms-2397790-supplementary.pdf]

Table S1. Expression levels of miR-21 and EGF before and after surgery.

Patient	Exosomal miR-21 (Ct)		EGF (pg/mL)		Clinical outcomes
	Pre-surgery	Post-surgery	Pre-surgery	Post-surgery	
1	29.31	30.11	38.04	34.28	Improving
2	27.63	26.34	14.01	39.6	Worsening
3	30.3	27.2	20.13	48.46	Worsening
4	32.2	28.91	14.31	50.43	Worsening
5	26.84	26.33	15.62	42.31	Worsening
6	28.76	29.59	111.85	32.14	Improving
7	29.78	30.05	24.98	20.35	Improving
8	30.01	29.99	3.91	5.18	Stationary
9	28.87	29.77			Improving
10	30.72	31.53			Improving
11	29.44	31.53			Improving
12	30.67	31.66			Improving
13	30	31.48			Improving
